# The Cannabinoid Receptor 2 Q63R Variant Modulates the Relationship between Childhood Obesity and Age at Menarche

**DOI:** 10.1371/journal.pone.0140142

**Published:** 2015-10-08

**Authors:** Giulia Bellini, Anna Grandone, Marco Torella, Emanuele Miraglia del Giudice, Bruno Nobili, Laura Perrone, Sabatino Maione, Francesca Rossi

**Affiliations:** 1 Department of Experimental Medicine, Pharmacology Division, Second University of Naples, Naples, Italy; 2 Department of Woman, Child and of General and Specialist Surgery, Second University of Naples, Naples, Italy; CHA University, REPUBLIC OF KOREA

## Abstract

**Background:**

The ovary is an important site where gene variants modulate pubertal timing. The cannabinoid receptor 2 (CB2) is expressed in the ovary, plays a role in folliculogenesis and ovulation, and can be modulated by estrogens. Obesity is strictly associated with early menarche and is characterized by sex hormone and endocannabinoid derangement.

**Aim:**

In this study, we investigated the role of the CB2 receptor in determining the age at menarche in obese girls.

**Methods:**

We studied a cohort of 240 obese girls (age 11.9±3 years; BMI z-score 2.8±0.8). The age at menarche (if it had already occurred) was recorded at the time of the visit or via phonecall. The *CNR2 rs*35761398 polymorphism, which leads to the CB2 Q63R variant, was detected by the TaqMan assay.

**Results:**

In total, 105 patients were homozygous for the R63-coding allele (RR), 113 were QR and 22 were QQ. Variance analysis revealed a significantly earlier age of menarche in subjects carrying the Q63 allele, which was also found after adjusting for BMI z-score (11±1.2 *vs*. 11.6±1.2 years, *p* = 0.0003). Logistic regression analysis demonstrated that patients homozygous for the Q allele had a 2.2-fold higher risk (odds ratio = 2.2; CI1.1–3.4; *p* = 0.02) of presenting with an early menarche (age at menarche <12 years).

**Conclusion:**

We demonstrated for the first time the association between the CB2 Q63R functional variant and the age at menarche in a cohort of Italian obese girls.

## Introduction

The age at menarche is a marker of pubertal timing in females. Pubertal timing is widely variable, a result of the interactions of both environmental and genetic determinants. The age at menarche is associated with obesity, type 2 diabetes, cardiovascular disease, breast cancer and all-cause mortality and is characterized by a complex genetic architecture [1–3]. The mechanisms that determine pubertal timing and underlie its links to disease risks remain unclear.

In the ovary, some gene variants such as the lin-28 homolog B (*LIN28B*) gene polymorphism have been strongly associated with the age at menarche, thus modulating pubertal timing [4,5]. Additionally, early menarche has been associated with childhood obesity [6].

Multiple endocrine pathways link childhood obesity to early pubertal onset [7–10].

The endocannabinoid (EC) system includes the EC endogenous lipid transmitters, their G-protein-coupled cannabinoid receptors type 1 and type 2 (CB1 and CB2), and the enzymes for EC biosynthesis and degradation. ECs are synthesized and immediately released to activate CB1 and CB2 “on demand”, although they can also accumulate in intracellular adiposomes [11].

Recent data suggest that the EC system plays a role in folliculogenesis and ovulation and is widely expressed in the ovarian medulla and cortex. The CB2 receptor is preferentially expressed with respect to CB1 in the granulosa cells (GCs) of follicles at all stages of development as well as in the corpus luteum and corpus albicans. CB2 stimulation induces follicle growth and oocyte maturation [12, 13]. Moreover, the EC system is disturbed in obesity, resulting in an enhanced tone that is associated with increased EC plasma levels [14].

Based on this evidence, we are tempted to speculate that a different CB2 receptor response could contribute to early menarche in obesity. Thus, we investigated a functional variant of the CB2 receptor, Q63R, for its potential influence on the age at menarche in a cohort of 240 Italian obese girls.

## Materials and Methods

### Subjects

We studied a cohort of 240 obese girls. Obesity was defined as a BMI exceeding the 95th percentile for age and sex. Patients with autoimmune disorders were excluded due to the previously described association between the CB2 Q63R variant and autoimmune disorders [15–18].

Assent from the girls and written informed consent from their parents was obtained. The study was conducted with the approval of the Ethic Committee of the Second University of Naples in compliance with the national legislation and the Declaration of Helsinki.

Assessment of menarchal age was obtained by the status quo method when recorded at the time of the visit if it had already occurred and by the recall or retrospective method via phone call if it had not yet occurred.

### Genotyping

Genomic DNA was extracted from peripheral whole blood with a DNA extraction kit (Roche Diagnostics, Branchburg, NJ, USA). Molecular screening for the *CNR2* rs35761398 polymorphism (CAA/CGG) underlying the CB2 Q63R substitution was performed using a TaqMan assay (Real Master Mix Probe, 5 PRIME, Germany). The primers and probes used were the following: sense 5'-GTGCTCTATCTGATCCTGTC-3' and anti-sense 5'-TAGTCACGCTGCCAATC-3'; AA-probe 5'-CCCACCAACTCCGC-3' and GG-probe 5'-CCCACCGGCTCCG-3' (PRIMM, Milan, Italy). Both PCR and post-PCR allelic discrimination were performed on a7900 HT Fast System Thermal Cycler (Applied Biosystems, Foster City, CA, USA).

Genotypes of random samples were confirmed by PCR followed by direct sequencing. The PCR program consisted of 94°C for 4 min followed by 31cycles of 94°C for 30 s, 60°C for 30 s and 72°C for 30 s. Primers were the following: forward 5'-GAGTGGTCCCCAGAAGACAG-3' and reverse 5'-CACAGAGGCTGTGAAGGTCA-3'. PCR products were sequenced using an ABI PRISM 9600 automated sequencer (Applied Biosystems, Foster City, CA, USA) and the Big Dye Terminator reaction kit (Applera, Foster City, CA, USA) according to the manufacturer’s instructions. All of the primers were chosen using Primer3 software (http://primer3.sourceforge.net/).

### Statistics

Differences between categorical variables were analyzed using a chi-squared test. A linear logistic regression was performed to analyze clinical data with respect to the CB2 Q63R variant. A *p* value < 0.05 was considered to be statistically significant. All of the analyses were performed using StatGraphics CENTURION XV.II (Adalta, Arezzo, Italy; STATPOINT TECHNOLOGIES INC., Virginia, USA).

## Results

We analyzed a cohort of 240 obese girls(age 12.9±2.6 years; BMI z-score 2.8±0.8) for the *CNR2* rs35761398 polymorphism (CB2 Q63R variant) by the TaqMan assay. Clinical data are summarized in Table 1.

**Table 1:** Clinical data of 240 obese Italian girls according to CB2 Q63R variant.

	RR	QR	QQ	*p*-value
**N** (%)	105 (43.8%)	113 (47%)	22 (9.2%)	
**Age** (years; mean ± SD)	13 ± 2.5	12.7 ± 2.7	13.8 ± 2.6	0.5
**z-score BMI** (mean ± SD)	2.9 ± 0.8	2.9 ± 0.7	3.1 ± 0.7	0.4
**W/Ht r** (mean ± SD)	0.62 ± 0.06	0.62 ±0.06	0.61 ± 0.05	0.2
**HOMA-IR** (mean ± SD)	5.5 ± 3.4	6 ± 4.5	5.8 ± 4.1	0.7
**Total Cholesterol** (mg/dl; mean ± SD)	157±27	155±27	164±32	0.35
**Triglycerides** (mg/dl; mean ± SD)	102± 63	102± 70	96 ± 45	0.9
**HDL** (mg/dl; mean ± SD)	42 ± 13	45± 9	43 ± 17	0.1
**Age at menarche** (years; mean ± SD)	11.6 ± 1.3	11± 1.3	11± 1	**0.0016**

SD: standard deviation; BMI: body mass index; W/ Ht r: waist/height ratio; *p*-values <0.05 have been considered significant and are shown in bold.

In total, 105 patients were homozygous for the R-encoding allele (R63), 113 were heterozygous (Q63R) and 22 were homozygous for the Q-encoding allele (Q63).

Allele frequencies were in Hardy Weinberg equilibrium (*p* = 0.28).

Variance analysis demonstrated a significantly earlier age of menarche in subjects carrying the Q allele also after adjusting for the BMI z-score (*p* = 0.0016). No differences in metabolic parameters were observed (Table 1). Logistic regression analysis demonstrated that Q63 subjects had a 2.05-fold higher risk of presenting menarche before 12 years of age (Odds Ratio (OR) = 2.05; CI 1.21–3.45; *p* = 0.0068).

**Fig 1:**
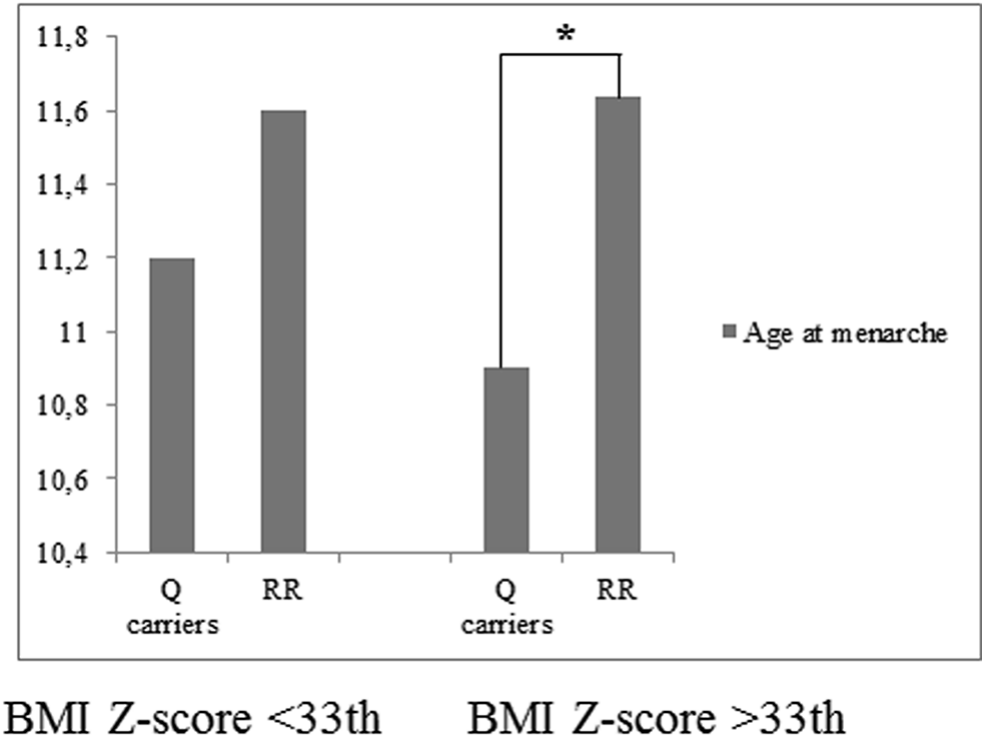
Differences in age at menarche according to BMI z-score tertiles. *p<0.001.

After stratifying by BMI z-score, the differences in the age at menarche among genotypes were not significant among patients in the lowest BMI z-score tertile (Fig 1).

## Discussion

In this study, we demonstrated, for the first time, an association between the *rs*35761398 polymorphism in the *CNR2* gene, which underlies the Q63R functional variant, and the age at menarche in a cohort of 240 Italian obese girls. CB2 differentially modulates its effector cells and downstream pathways depending on the presence of glutamine or arginine at codon 63 of the N-terminal domain; thus, the Q63 variant is more functional than the R63 mutant. Indeed, lymphocytes derived from RR-subjects display minor proliferation inhibition compared with those derived from QQ subjects [15, 19].

Our data reveal that the CB2 Q63 variant is associated with an earlier age of menarche in obese girls, and there was a significantly higher probability of menarche before 12 years of age.

Interestingly, the *CNR2* gene, which encodes for CB2, maps to 1p36, which is a region that displays multipoint LOD scores greater than 1.00 in the Weight-Adjusted Genome Scan Analysis for Mapping Quantitative Trait Loci for Menarchal Age, although genome wide scan association studies have never identified the specific contribution of *CNR2* [20]. Thus, the possibility exists that the effect exerted by the Q63R variant on the age at menarche is not detectable in women of normal weight, while it could be unmasked by facilitating environmental factors such as obesity. Consistently, by dividing patients into three different subgroups according to three different BMI ranges, we observed that the association persists only in the subgroup with the highest BMI range.

Common features of childhood obesity are elevated leptin levels and insulin resistance. Leptin, which is a hormone released by adipocytes, plays a major role in puberty initiation and progression by directly and positively acting on the hypothalamic Gonadotropin Releasing Hormone (GnRH) secretory system. Moreover, leptin receptors have been identified in both GCs and theca cells (TCs) of follicles [21]. Although we have not measured leptin levels in our cohort, leptin levels are high in obese subjects. Leptin also downregulates orexigenic factors, which include ECs [22]. Therefore, whereas the leptin signaling is uniformly high in obesity, it is possible that the CB2 receptor will better respond to the decreased EC levels when the functional variant Q63 is expressed.

Alternatively, it is plausible that the leptin resistance that accompanies obesity could mask the effect of leptin in terms of EC tone.

Insulin acts on various organs including the ovary and adipocytes to increase sex steroid bioavailability and to decrease sex hormone binding levels [23]. Thus, elevated sex steroid levels in obese prepubertal children could early activate hypothalamic-pituitary puberty. Interestingly, CB2 expression can be modulated by estrogens. In particular, 17β-estradiol increases CB2 expression, possibly by the recruitment of a putative estrogen responsive element (ERE) in the *CNR2* gene [24]. Thus, the increased levels of estrogens in obese girls could enhance CB2 expression also in the follicles and could be a more efficient trigger of their maturation if the Q63 is present.

Finally, much evidence demonstrates that the EC tone is higher in obese subjects [14, 22]. Thus, the increased EC levels as well as the accompanying obesity could further stimulate the ovary through the CB2 receptor and predispose females to early menarche, whereby a more functional variant is present and more responsive.

All of these mechanisms might be enough to induce an early response of the follicles as a peripheral target, and in turn, early menarche onset.

Although CB2 receptor stimulation induces follicle growth and oocyte maturation[12], very little is known about the mechanism underlying this process.

It is noteworthy that CB2 receptor stimulation by selective agonists activates the serine/threonine protein kinase Akt signaling pathway, which is involved in cell growth, survival, proliferation and metabolism. This CB2-mediated activation prevents apoptosis of cardiac cells during ischemia/reperfusion and of central neurons after axonal damage as well as accelerates *in vitro* oligodendrocyte differentiation [25–28].

Several lines of evidence indicate that the Akt signaling pathway is a critical regulator of follicle growth, differentiation and survival, which determines the pool of primordial follicles and the transition from the quiescent to the growing phase, modulating the granulosa cell apoptosis throughout folliculogenesis and regulating the spindle organization in oocytes during meiosis resumption [29–32]. Moreover, suppression of Akt activity determines a delayed resumption of meiosis in mouse oocytes, and female mice deficient in the Akt1 isoform demonstrate reduced fertility and dysregulation of follicular and oocyte development, including a delay in estrus onset [33].

In conclusion, we hypothesize that the CB2 receptor may be part of the peripheral pathway that mediates the ovarian response to peripheral estrogens, possibly through the Akt pathway. In obese girls, all the permissive signals for puberty are dysregulated (such as estrogens, ECs and leptin), and the more functional CB2 receptor variant (Q63) could enhance ovarian stimulation, thus contributing to early menarche by affecting either puberty initiation or the rate of puberty progression. These data may add a new piece to the complex puzzle of female puberty regulation. Our findings could moreover help to identify obese patients who have an increased risk of developing early menarche as well as to individualize treatment and follow-up.
